# Evaluation of the insulin administration technique in a tertiary hospital

**DOI:** 10.1186/1758-5996-7-S1-A176

**Published:** 2015-11-11

**Authors:** Karla Borges Daniel, Karina Saiuri Takatori, Adriana Russo Fiore, Arnaldo Moura Neto, Elizabeth João Pavin, Walter José Minicucci, Maria Cândida Ribeiro Parisi

**Affiliations:** 1 UNICAMP, Campinas, Brazil

## Background

Optimizing glycemic control is important to minimize the risk of macro and microvascular complications in diabetes. Therefore, it is important that patients under insulin treatment know the correct technique for insulin self-administration to ensure proper management.

## Objective

Assess whether patients with longstanding diabetes followed in a tertiary hospital know how to correct self-administer their insulin.

## Materials and methods

Cross sectional study consisting on the application of a questionnaire about the procedures of insulin self-administration to 100 patients treated at a tertiary type 2 diabetes mellitus unit. The questions assessed time of diabetes, types of insulin used, total insulin dose, comorbidities and use of other drugs. There were also specific questions about the technique of insulin self-administration according to guidelines of the Brazilian Diabetes Society.

## Results

Of 100 patients evaluated, 50% were female, mean age 61.54 yrs. (range 35 – 86 yrs.). The mean disease duration was 18,52 yrs. (2 – 40 yrs.). Most patients learned how to apply insulin with a nurse (48%), 17% were instructed by a doctor and 27% could not remember. All but 2 patients used drugs other than insulin. As for type of insulin, 80 patients (80%) used human insulin, most of them (61%) three injections a day and 76% mixed the two types of insulin in the same syringe. The 100 cc, 50cc and 30cc syringes were used by 59%, 27% and 5% of patients, respectively, reflecting the greater distribution of 100 cc syringes by basic health units. The mean total insulin dose was 77.72 IU (8-212 IU). Regarding the 20 specific questions on insulin administration technique, the mean number of correct answers was 9.87 (6 – 13).

## Conclusion

This study shows that even in a tertiary hospital there is a high rate of mistakes in insulin self-administration, which may be associated with poor glycemic control and an increased incidence of diabetes complications, including hypoglycemia. Thus, it is important that all health care professional actively inquire how the patients routinely administer their insulin, since diabetes is a chronic disease that requires a continuous educational process

**Figure 1:**
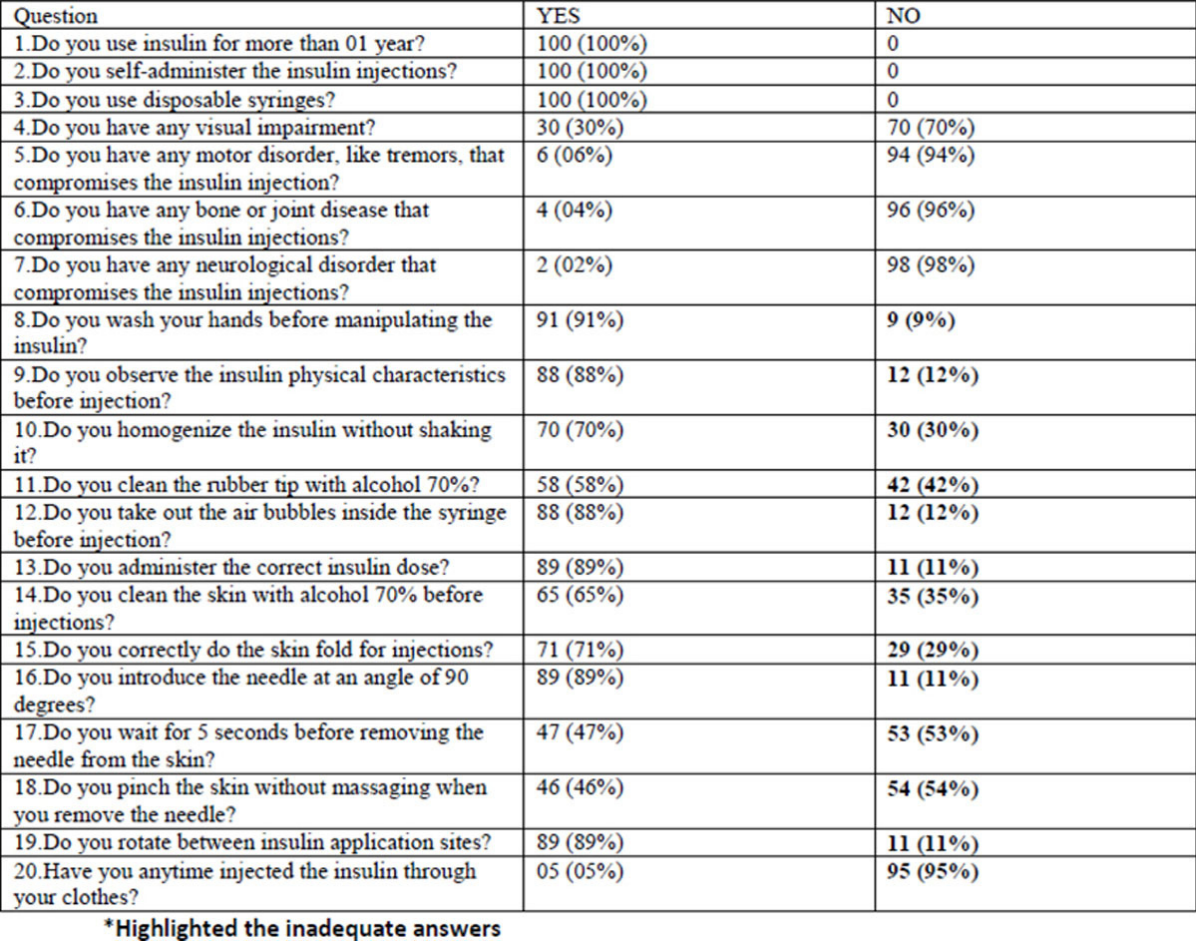
Results of the questionnaire.

